# Predicting efficacy and guiding procedure choice in non-vascularized bone grafting: a CT Radiomics and clinical predictor approach

**DOI:** 10.1186/s12891-023-07095-1

**Published:** 2023-12-11

**Authors:** Hao Chen, Peng Xue, Hongzhong Xi, Shuai He, Guangquan Sun, Xin Liu, Bin Du

**Affiliations:** Affiliated Hospital of Nanjing University of Chinese Medicine, Nanjing, Jiangsu 210029 China

**Keywords:** Osteonecrosis of femoral head, CT radiomics, Non-vascularized bone grafting, Hip preservation

## Abstract

**Objectives:**

There is no practical approach for accurately predicting the efficacy of non-vascularized bone grafting (NVBG) and guiding its optimal procedure.

**Materials and methods:**

This study enrolled 153 patients with 182 hips that underwent NVBG procedures. The patients were randomly divided into a training cohort (n = 130) and a validation cohort (n = 52). In the training cohort, radiomics model, clinical model, and combined radiomics-clinical (C-R) model were constructed using Rad-scores and clinical predictors to predict the efficacy of NVBG. The optimal model was visualized by a nomogram and assessed by decision curve analysis (DCA). 128 hips that underwent successful NVBG were then randomized into a new training cohort (n = 92) and a new validation cohort (n = 36), and three models were constructed and validated to predict the choice of NVBG procedure.

**Results:**

Japanese Investigation Committee (JIC) classification, exposure to risk factors postoperative, and Rad-scores consisting of four radiomics features were independent predictors for the efficacy of NVBG (*P* < 0.05). The C-R model provided better performance in both the training cohort (AUC: 0.818) and validation cohort (AUC: 0.747). To predict the choice of NVBG procedure, the C-R model built by JIC classification and Rad-scores consisting of five radiomics features showed the finest performance in both cohorts (AUC: 0.860 and 0.800, respectively). DCA showed great benefit using the C-R model for the choice of NVBG procedure.

**Conclusion:**

The approach integrated by CT radiomics and clinical predictors can be visually and quantitatively applied to predict the efficacy and guide the choice of NVBG procedure with great predictive accuracy.

**Supplementary Information:**

The online version contains supplementary material available at 10.1186/s12891-023-07095-1.

## Introduction

Osteonecrosis of the femoral head (ONFH) is a rapidly progressive and debilitating disease [1] with an increasing annual incidence worldwide [2–5], affecting over 20 million people [6]. Despite identification of several risk factors, including trauma [7], corticosteroid use [8], excessive alcohol consumption [9], and smoking [10], the etiology and pathogenesis of ONFH remain unclear, leading to a lack of effective prevention initiatives. Total hip arthroplasty (THA) is the exclusive reliable option for end-stage osteoarthritis resulting from ONFH [7, 11]. Due to the complications of THA and the deficiencies of prosthesis durability [12, 13], hip preservation surgery has high clinical and social value for young and middle-aged patients in the early stages of ONFH to delay initial THA [7, 11, 14].

Non-vascularized bone grafting (NVBG) is a viable treatment for pre- and early post-collapse ONFH [15, 16], with the Phemister procedure and lightbulb procedure being two classic and distinct NVBG procedures to deposit non-vascularized bone into the necrotic lesion within the femoral head [3, 11, 17, 18]. Although NVBG has a high success rate, numerous failures still occur due to unsuitable patient selection and the choice of an inappropriate NVBG procedure [19, 20]. Currently, treatment strategies for hip preservation require comprehensive consideration of patients’ clinical and radiographic data, including CT imaging, which accurately indicates the osteology of the structures such as the size of the necrotic area, extent of the sclerotic region, and other information crucial for making informed treatment decisions [21–23]. However, there is no consensus among orthopedic surgeons regarding the operative management of patients who undergo NVBG, and subjective variation in diagnosing the staging, site, and extent of necrotic areas can cause differences in surgical outcomes. Extracting, quantifying, and utilizing deep image information directly with the naked eye remains difficult, underscoring the need for practical approaches that guide the optimal selection of NVBG procedures and predict their efficacy with greater accuracy.

The concept of radiomics has emerged as a promising approach to medical imaging with the rapid development of medical artificial intelligence technology [24]. Radiomics captures distinct phenotypic differences in target regions from diagnostic images using algorithms or statistical analysis tools, providing massive information beyond visual analysis and sensitively identifying subtle heterogeneity in morphology and function between different parts. While radiomics has been widely used in the field of diagnosis and prognosis [25–27], few studies have applied radiomics analysis in hip preservation despite its powerful potential.

Our institution has been performing NVBG using the Phemister procedure and lightbulb procedure, with a large number of cases, detailed clinical and radiographic data, and medium to long-term follow-up. Therefore, we developed a visual and quantitative assessment approach using data from patients and evaluated its performance internally. Our study aims to provide valuable insights into the optimal selection and efficacy of NVBG procedures for hip preservation using CT radiomics and clinical predictors. We seek to determine suitable patients for hip preservation with NVBG, to identify the most appropriate procedure, and to explore strategies to improve the success rate of hip preservation.

## Materials and methods

### Patients

A total of 175 patients (216 hips) with pathologically confirmed ONFH were enrolled in this study from June 2009 to June 2019. Inclusion criteria were as follows: (1) the surgical procedure was NVBG (Phemister procedure or lightbulb procedure) performed by the same surgeon; (2) no history of other hip preservation surgical treatment; (3) completeness and availability of clinical and radiographic data; and (4) follow-up duration greater than three years. Exclusion criteria were: (1) insufficient CT image quality for radiomics analysis; and (2) postoperative cancer, hip tumor, bone tuberculosis, and other disorders. The hip was used as a unit, and clinical data were recorded twice for cases with bilateral hip preservation. THA or Harris scores < 90 with progressive collapse of the femoral head on imaging within three years after NVBG were defined as failure. Survival rate and survival time were calculated using June 2022 as the follow-up endpoint.

**Fig. 1:**
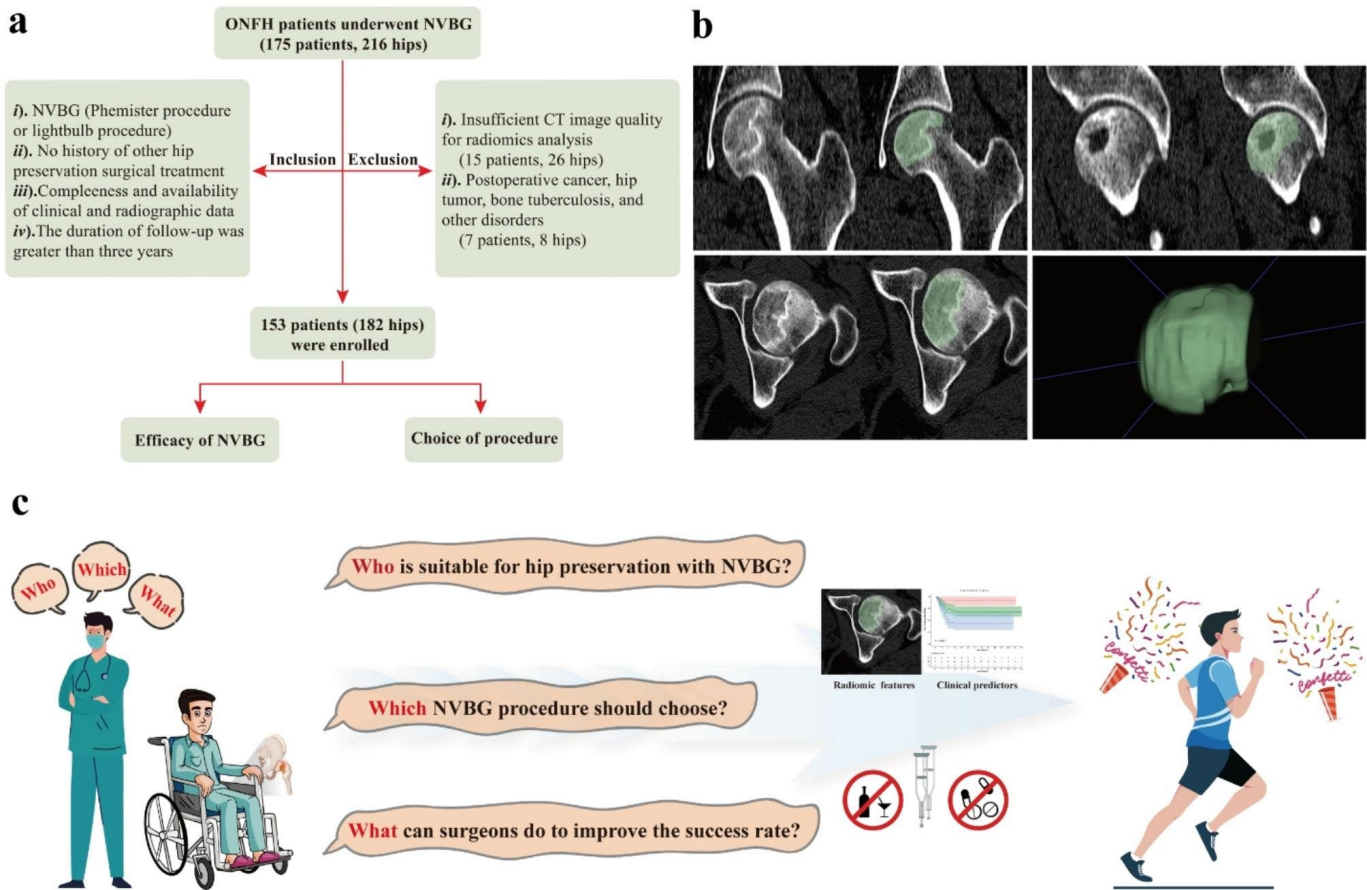
**a** Flowchart of study enrollment. **b** Schematic diagram of ROI segmented and outlined in coronal, sagittal and axial positions by manual mode in the bone window. 3D ROI of necrotic area generated from them. **c** Schematic illustrations of study flowchart for predicting efficacy and choice of NVBG procedures by analyzing CT radiomics features and clinical predictors

This study was registered in the **** Clinical Trial Registry (Registration ID: ChiCTR2300067945, 01/02/2023), and approved by the ethics committee of **** (Ethical approval ID: ***), which waived the requirement for individual consent due to the use of retrospective data. The case selection process is shown in Fig. 1a, and the flowchart of the study is presented in Fig. 1c.

### Clinical data

Clinical data associated with the efficacy of hip preservation, as reported in the literature [3, 7–10, 18, 28], were recorded. This included general data such as age, gender, affected side, disease duration, BMI, and etiology. Preoperative examination indexes were also recorded, including D-dimer, alkaline phosphatase (ALP), white blood cell count (WBC), neutrophil percentage (N), and fucosidase(AFU). Additionally, ARCO stage, JIC classification, and preoperative Harris scores were documented.

### CT image acquisition

All patients underwent preoperative CT scans of bilateral hips, with images acquired from the PACS system in DICOM format. CT scans were performed using a Brilliance CT scanner (128-row, Philips Healthcare, Netherlands) or a LightSpeed VCT scanner (64-row, GE Healthcare, USA), with scan parameters including tube voltage of 120-140 kV, tube current of 220-680 mA, exposure time of 250–800 mS, layer thickness of 1.0–3.0 mm, layer spacing of 1.0–3.0 mm, and matrix sizes of 512 × 512.

The resampling process was carried out using the bilinear interpolation method, with the resampling layer thickness and layer spacing set at 1 mm. Images were then imported into 3D Slicer (https://www.slicer.org, V5.0.2) in *nii* format. Values of extracted radiomics features were normalized using the Z-transform method.

### ROI segmentation and feature extraction

The zone with abnormal density, such as osteosclerosis, cystic change, and subchondral bone fracture, is defined as the region of interest (ROI). These ROIs were manually segmented and outlined in coronal, sagittal, and axial positions in the bone window (window width 1500HU, window level 400U) by a senior radiologist (**) and a senior orthopedic surgeon (**) to produce 3D ROIs (Fig. 1b). The Pyradiomics plug-in was used to automatically extract 851 radiomics features from each ROI. The intra-class correlation coefficient (ICC) was used to assess the consistency of radiomics features extracted by the two doctors, a consistency score of > 0.75 was considered not susceptible to interobserver variation, and the corresponding features were retained.

### Construction and application of models for the efficacy of NVBG

To determine who is suitable for hip preservation with NVBG, we constructed predictive models for the efficacy of NVBG. The hips were randomly divided into a training cohort (n = 130) and a validation cohort (n = 52) at a ratio of 7:3 using computer-generated random numbers to ensure that there was no overlap of hips in the two datasets. In the training cohort, clinical data were analyzed by univariate and multivariate analyses to screen for clinical predictors. The least absolute shrinkage and selection operator (LASSO) method was used to select the most useful radiomics features for prediction, and the radiomics score (Rad-scores) was calculated for each patient through a linear combination of selected features weighted according to their respective coefficients.

Three models were constructed in the training cohort based on the extracted features: the Clinical model, Radiomics model, and C-R model based on the combination of clinical predictors and Rad-scores. The predictive performance of the three models was evaluated and validated in both cohorts through the receiver operating characteristic curve (ROC), area under curve (AUC), and DeLong test to compare the AUCs. The predictive performance of each model was assessed based on AUC, sensitivity, and specificity, while the goodness of fit of each model was evaluated using a calibration curve with Hosmer-Lemeshow test. To provide clinicians with a quantitative approach for assessing the efficacy of NVBG, we visualized the optimal model by constructing a nomogram and estimated the clinical utility using Decision Curve Analysis (DCA). DCA was performed by calculating the net benefits for a range of threshold probabilities in both the training and validation cohorts.

### Construction and application of model for the choice of NVBG procedures

To determine which NVBG procedure should be chosen, we constructed predictive models for the choice of NVBG procedures. Of the 128 hips that underwent successful NVBG, they were divided into a new training cohort (n = 92) and a validation cohort (n = 36) at a ratio of 7:3 to ensure that there was no overlap of hips in the two datasets. Radiomics features and clinical predictors were extracted from patients, with the choice of NVBG procedures used as the dependent variable. The predictive models were then constructed and validated, with the optimal model visually quantified to assist in clinical procedure selection.

### Statistical analysis

All statistical tests were performed using SPSS 26.0 and R statistical software (version 1.2.5042, https://www.r-project.org/). The differences in relevant baseline clinical data between the training and validation cohort were assessed using independent sample t-test, *χ*^*2*^ test, or Mann-Whitney U test by SPSS 26.0, as appropriate. Kaplan-Meier survival estimate analysis was used to evaluate the value of Rad-scores and clinical predictors. All statistical analyses were two-sided and evaluated with *P* < 0.05 considered statistically significant.

In R statistical software, we used the “glmnet” package to peform the LASSO regression analysis, while the survival curves were obtained using the “survminer” package. ROC curves were plotted using the “pROC” package, and calibration plots were constructed using the “resourceSelection” package. The nomogram was constructed with the use of the “rmda” package, while DCA was performed using both the “rmda” and “ggDCA” packages.

## Results

### Patients’ characteristics

Initially, 153 patients with a total of 182 hips were enrolled in the study (Table S1 & Table S2). Univariate analyses indicated that there was no significant difference between the clinical characteristics of the two groups of patients in their respective training and validation cohorts (P > 0.05), indicating that patients had a balanced distribution of baseline clinical characteristics.

**Fig. 2:**
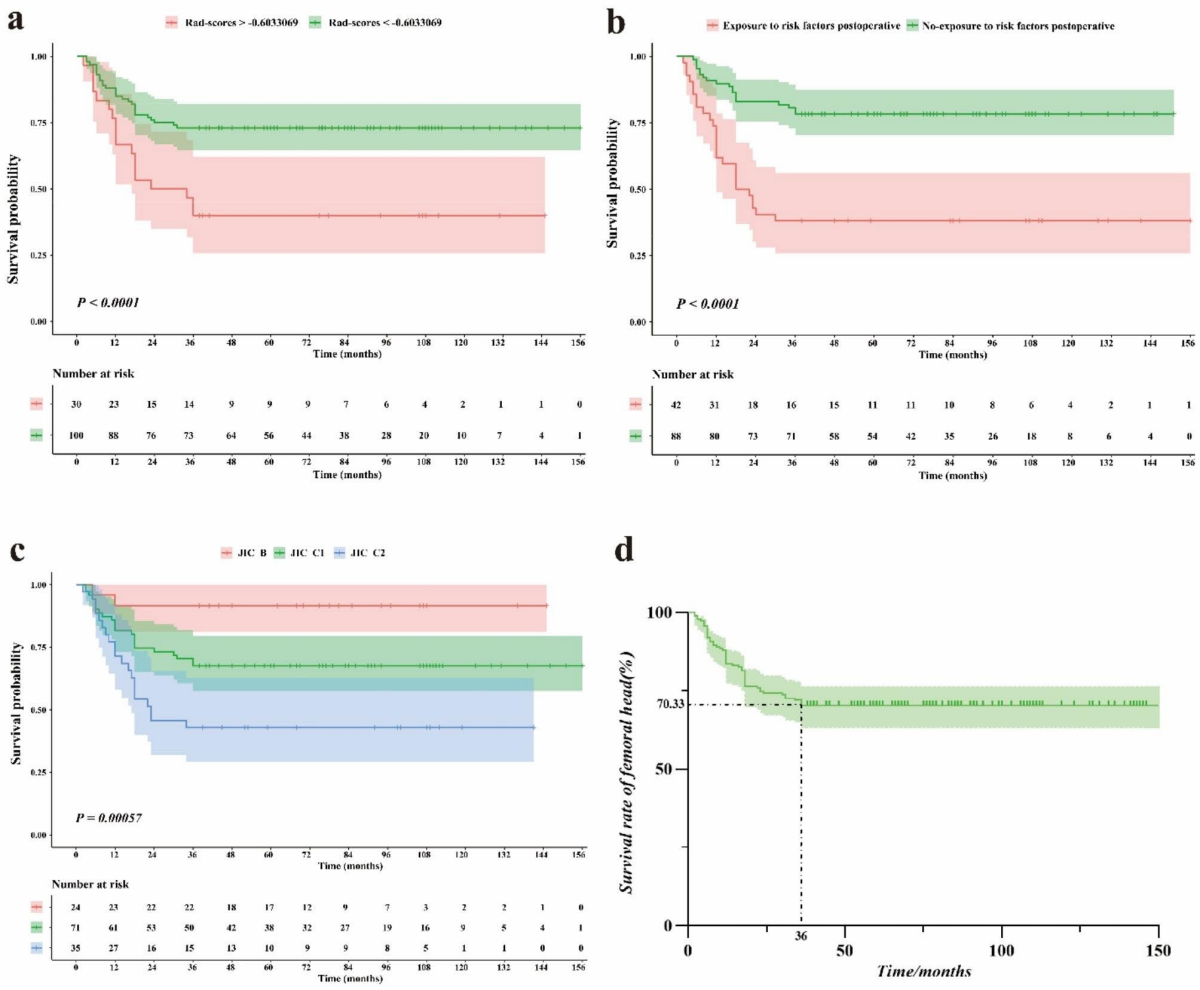
**a**-**c** Kaplan-Meier analyses of the three selected features for patients in the training cohort. **d** Survival rate of femoral head over time

At the 3-year postoperative follow-up, 128 hips had a satisfactory curative effect, resulting in a success rate of 70.33%. The Kaplan-Meier survival curve showed a flat progression in survival time postoperatively, with femoral survival essentially stable at 36 months (Fig. 2d). At the final follow-up, 120 hips were successful, with a mean survival time of (65.56 ± 43.27) months and a hip preservation success rate of 65.93%.

### Feature importance & performance of models for the efficacy of NVBG

**Fig. 3:**
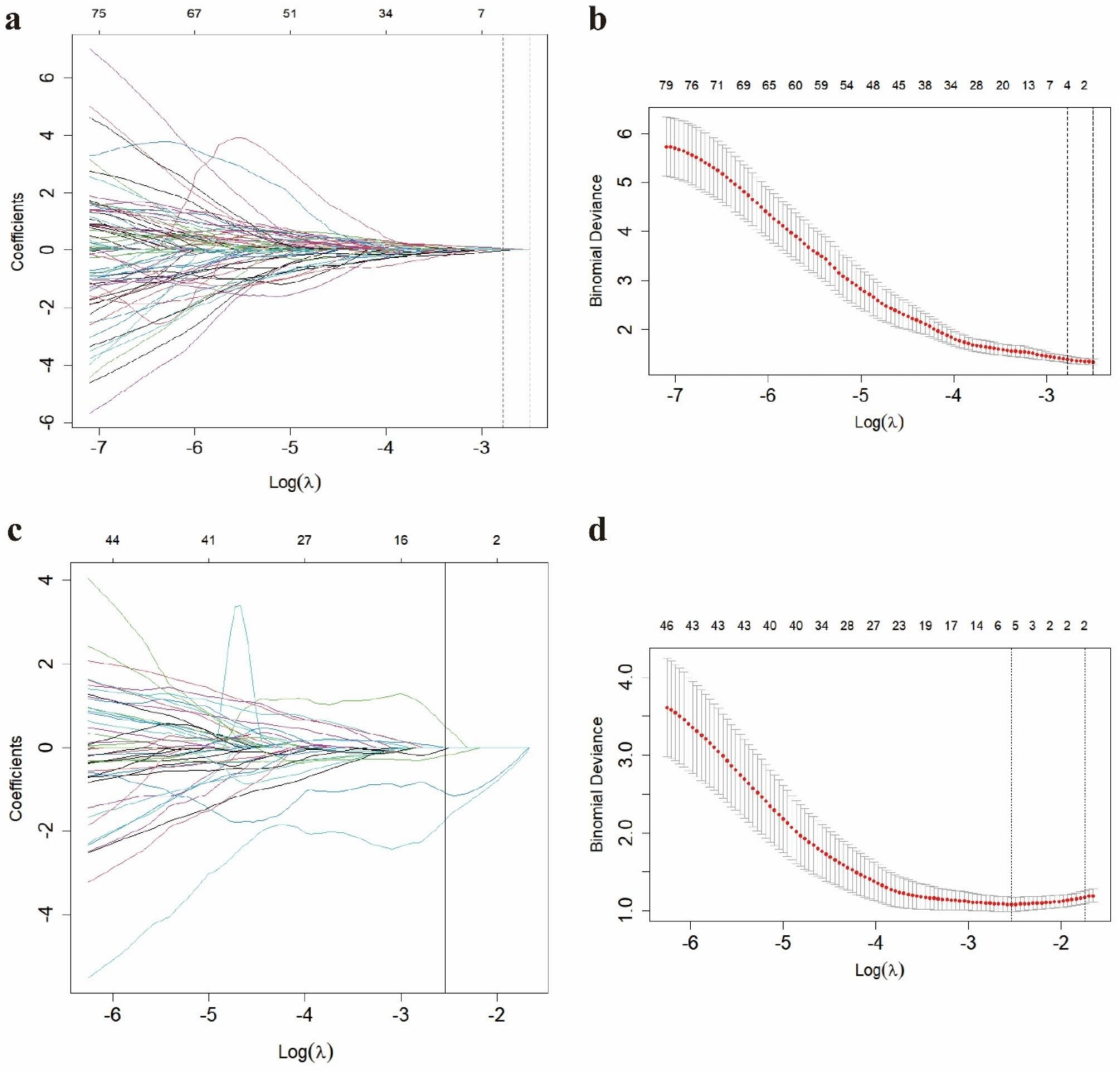
Radiomics features selected by LASSO to determine the efficacy of NVBG (**a**-**b**) and the choice of NVBG procedure (**c**-**d**). (**a, c**) Regression coefficient plot, (**b, d**) Cross-validation plot

There were statistically significant differences in JIC and exposure to risk factors postoperatively (Table 1, *P* < 0.05), which were independent clinical predictors used to construct the clinical model. After excluding 147 features with poor interobserver consistency following ICC analysis, a total of 704 radiomics features were retained and averaged. Based on the results of LASSO analysis (Fig. 3a-b), three original features (shape_Elongation, firstorder_InterquartileRange, GLCM_JointAverage) and one wavelet feature (LHH_firstorder_Mean) were most closely associated with the efficacy of NVBG and were used to calculate Rad-scores. Rad-scores were calculated as follows: Rad-scores=-0.637561473-0.01744×original_shape_Elongation + 0.017267×original_firstorder_InterquartileRange + 0.04948×original_glcm_JointAverage + 0.065846×wavelet-LHH_firstorder_Mean. Kaplan-Meier analysis showed that patients with a Rad-score greater than − 0.6033069, exposure to risk factors postoperatively, and types C2 of JIC classification had a significantly higher failure rate of NVBG (*P* < 0.0001, Fig. 2a-c).

**Table 1:** Univariate and multivariate analysis for efficacy of NVBG in the training cohort

Variables	Univariate Analysis			Multivariate Analysis		
	HR	95%CI	P	HR	95%CI	P
Gender	0.88	0.436–1.777	0.721			
Age	1.027	1.001–1.053	0.042			
Affected side	0.58	0.312–1.078	0.085			
Etiology	1.033	0.761–1.403	0.834			
Disease duration	1.004	0.963–1.048	0.838			
Exposure	0.251	0.139–0.456	**< 0.001**	0.309	0.168–0.565	**< 0.001**
ARCO stage	1.577	1.010–2.462	0.068			
JIC classification	2.474	1.539–3.976	**< 0.001**	2.118	1.288–3.483	**0.003**
BMI	1.027	0.919–1.148	0.640			
Harris preoperative	0.989	0.962–1.017	0.435			
D-dimer	1.122	0.897–1.402	0.313			
N	0.995	0.967–1.024	0.730			
ALP	0.996	0.983–1.008	0.474			
AFU	0.985	0.945–1.026	0.458			

**Fig. 4:**
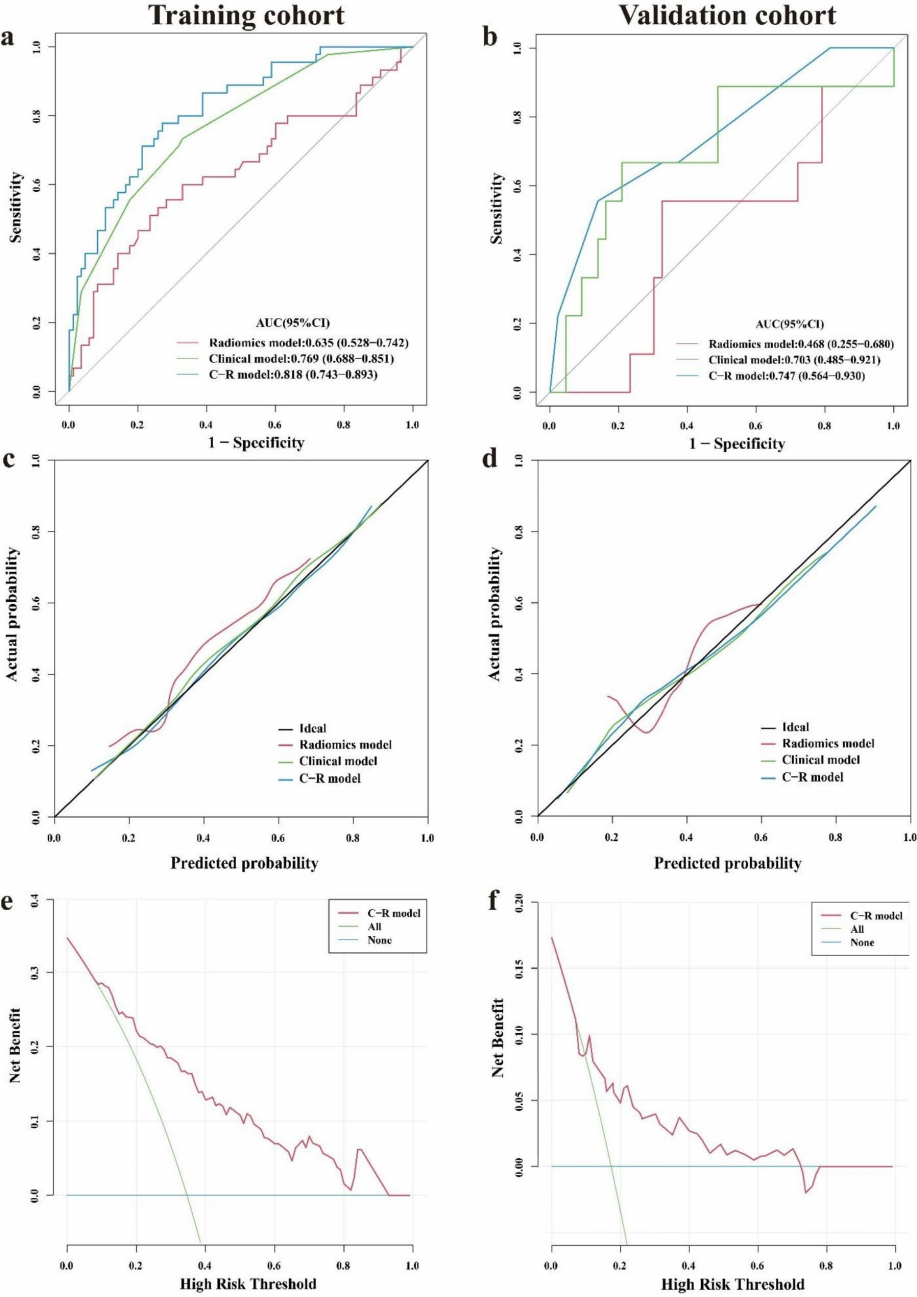
ROC curves (**a**-**b**) and calibration curves (**c**-**d**) for radiomics model, clinical model and C-R model for predicting the efficacy of NVBG in the training and validation cohort. Decision curve analysis for C-R model in training cohort (**e**) and validation cohort (**f**)

In the training cohort, the C-R model had an AUC of 0.818 (95% CI: 0.743–0.893), predicting sensitivity of 0.778 and specificity of 0.729 at the optimum cut-off of 0.308. In the validation cohort, the AUC of C-R model was 0.747 (95% CI: 0.564–0.930), with a predicting sensitivity of 0.556 and specificity of 0.860 at the optimum cut-off of 0.480 (Fig. 4a-b & Table 2), which was superior to other models (*P* < 0.05). The Hosmer-Lemeshow test showed that the C-R model had a superior goodness of fit in both the training cohort (P = 0.988) and validation cohort (P = 0.787), and the calibration curves of the C-R model demonstrated good agreement between prediction and observation in both cohorts as well (Fig. 4c-d).

**Table 2:** Performance of three models for predicting efficacy of NVBG

Cohorts	Models	AUC	Cutoff point	Sensitivity	Specificity	95%CI	
						Lower	Upper
Training cohort	Radiomics model	0.635	0.356	0.511	0.765	0.528	0.742
	Clinical model	0.769	0.245	0.733	0.671	0.688	0.851
	C-R model	0.818	0.308	0.778	0.729	0.743	0.893
Validation cohort	Radiomics model	0.468	0.338	0.556	0.674	0.255	0.680
	Clinical model	0.703	0.477	0.667	0.791	0.485	0.921
	C-R model	0.747	0.480	0.556	0.860	0.564	0.930

**Fig. 5:**
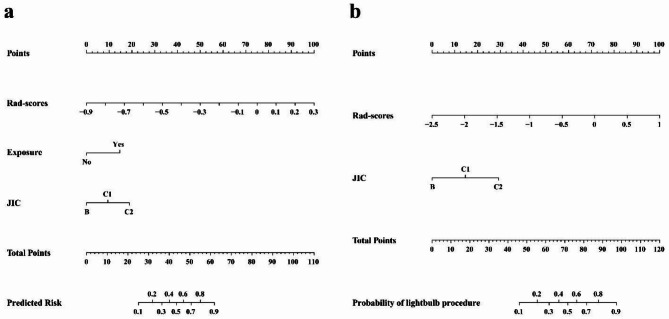
Nomograms of the C-R model for predicting the efficacy of NVBG (**a**) and the choice of NVBG procedures (**b**)

A nomogram was developed to visualize the C-R model (Fig. 5a), which revealed that patients with lower Rad-scores, no further exposure to risk factors postoperatively, and types B and C1 of JIC classification had a lower risk of failure postoperatively in NVBG. Decision curve analysis for the C-R model (Fig. 4e-f) showed that if the threshold probability of a patient or doctor is > 10%, using the C-R model to predict efficacy adds more benefit than either the treat-all-patients scheme or the treat-none scheme.

### Feature importance & performance of models for the choice of NVBG procedures

JIC classification was found to be an independent clinical predictor associated with the choice of procedures (Table 3, *P* < 0.05). Five wavelet features (LHL_firstorder_Mean, HHL_firstorder_Mean, LHL_firstorder_Median, LLL_glcm_MCC, HHH_gldm_DependenceNonUniformityNormalized) were proven to be most useful for predicting the choice of NVBG procedures (Fig. 3c-d), and Rad-scores were calculated as follows: Rad-scores=-0.867190064-1.09262×wavelet-LHL_firstorder_Mean − 1.7387×wavelet-LHL_firstorder_Median + 0.487119×wavelet-HHL_firstorder_Mean − 0.05207×wavelet-HHH_gldm_DependenceNonUniformityNormalized − 0.19791×wavelet-LLL_glcm_MCC.

**Table 3:** Univariate and multivariate analysis for the choice of NVBG procedures in the training cohort

Variables	Univariate Analysis			Multivariate Analysis		
	HR	95%CI	P	HR	95%CI	P
Gender	1.273	0.393–4.117	0.687			
Age	0.971	0.932–1.012	0.164			
Affected side	1.210	0.481–3.040	0.686			
Etiology	1.073	0.677–1.699	0.764			
Disease duration	0.964	0.875–1.061	0.451			
Exposure	0.505	0.170–1.497	0.218			
ARCO stage	1.115	0.441–2.820	0.818			
JIC classification	3.039	1.380–6.692	**< 0.001**	2.742	1.276–6.356	**0.006**
BMI	0.878	0.728–1.058	0.172			
Harris preoperative	0.979	0.934–1.027	0.387			
D-dimer	1.032	0.627–1.697	0.901			
N	1.039	0.982-1.100	0.182			
ALP	1.009	0.992–1.025	0.300			
AFU	1.019	0.961–1.080	0.532			

**Fig. 6:**
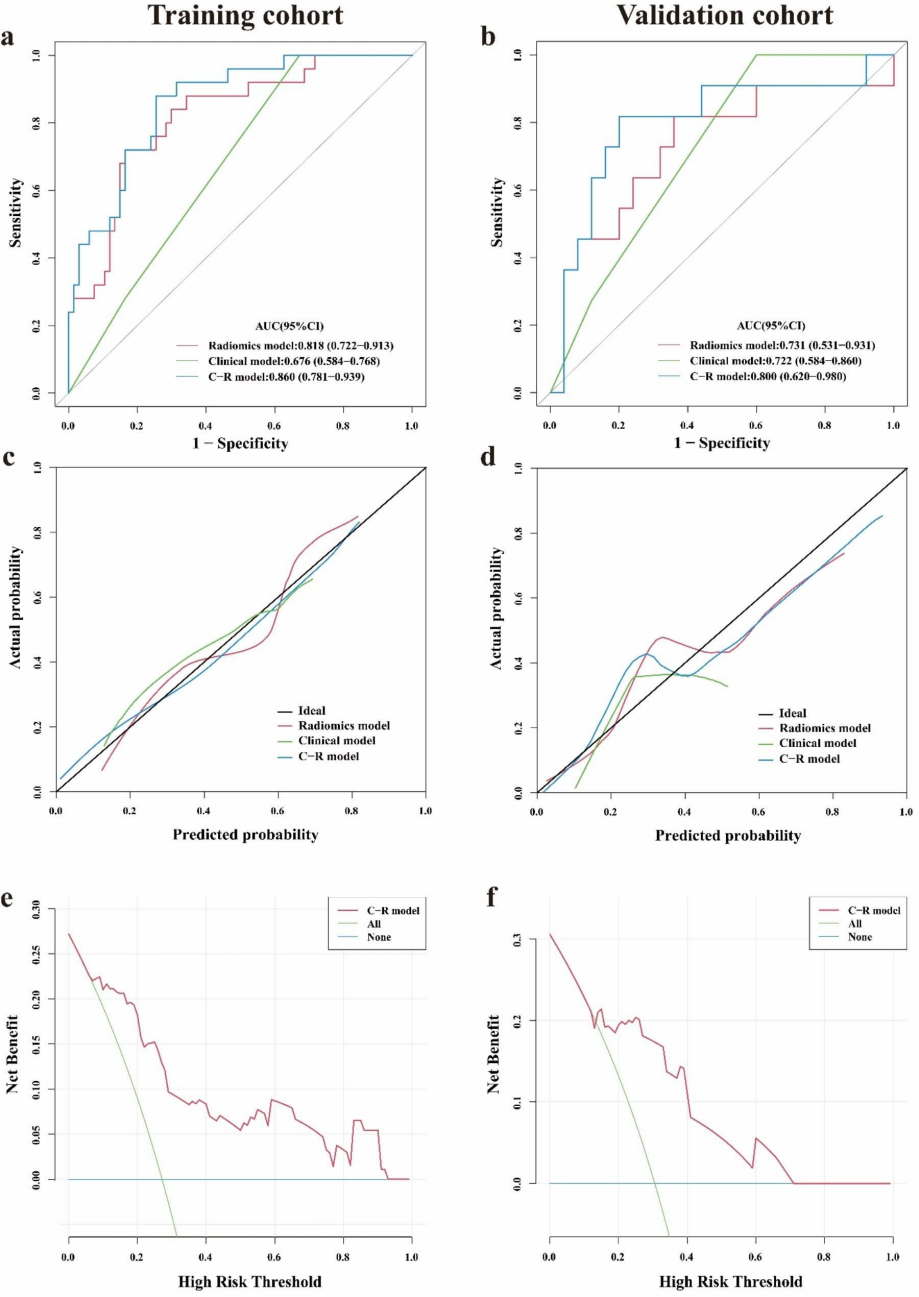
ROC curves (**a**-**b**) and calibration curves (**c**-**d**) for radiomics model, clinical model and C-R model for predicting the choice of NVBG procedures in the training and validation cohort. Decision curve analysis for C-R model in training cohort (**e**) and validation cohort (**f**)

Compared to the other two models, the C-R model had superior prediction performance, with an AUC of 0.860 (95%CI, 0.781–0.939) in the training cohort and 0.800 (95% CI, 0.620 to 0.980) in the validation cohort (*P* < 0.05) (Table 4; Fig. 6a-b). The Hosmer-Lemeshow test showed that the C-R model had a good goodness of fit in both the training cohort (P = 0.727) and validation cohort (P = 0.195). The calibration curve of the C-R model demonstrated good agreement between prediction and observation in the training cohort (Fig. 6c-d), while deviated calibration was observed in the validation cohort.

**Table 4:** Performance of three models for predicting the choice of NVBG procedures

Cohorts	Models	AUC	Cutoff point	Sensitivity	Specificity	95%CI	
						Lower	Upper
Training cohort	Radiomics model	0.818	0.309	0.720	0.836	0.722	0.913
	Clinical model	0.676	0.181	1.000	0.328	0.584	0.768
	C-R model	0.860	0.242	0.778	0.746	0.781	0.939
Validation cohort	Radiomics model	0.731	0.185	0.818	0.640	0.531	0.931
	Clinical model	0.722	0.181	1.000	0.400	0.584	0.860
	C-R model	0.800	0.261	0.818	0.800	0.620	0.980

The nomogram of the C-R model showed that patients with higher Rad-scores and types C1 and C2 of JIC classification should choose the lightbulb procedure, whereas patients with lower Rad-scores should choose hip preservation with the Phemister procedure (Fig. 5b). Decision curve analyses showed that the total net benefit of the C-R model trended similarly in both the training and validation cohort, and using the C-R model for the choice of NVBG procedures resulted in greater benefit if the threshold probability was greater than 15% (Fig. 6e-f).

## Discussion

Currently, there is a general consensus in the academic community on the criteria for assessing the efficacy of hip preservation [18, 28–31]. However, there is no definitive answer to the question of the status of the femoral head postoperatively that defines its success. Previous studies have used the outcome at the last follow-up to assess efficacy, which may be inappropriate. For most patients, the primary goal of undergoing hip preservation is to delay initial THA, rather than avoid it entirely. It is demanding to use the result of the final follow-up to assess efficacy, as many patients have achieved longer survival of the femoral head after hip preservation before THA. Furthermore, a non-weight bearing period of three months is required postoperatively, and a short-term follow-up cannot reflect patient satisfaction with the treatment. Thus, choosing the right point in time is important in assessing the efficacy of hip preservation and determining whether a patient is suitable for surgery. In this study, the majority of failure cases occurred between 2 and 3 years postoperative, with only 8 hips failing after 3 years. The survival time of the femoral head after 3 years was relatively stable, and the success rate of NVBG was comparable to that at the final follow-up. Therefore, we chose the status of the femoral head at the 3-year postoperative as the basis for assessing efficacy and constructed predictive models to identify patients suitable for NVBG and select appropriate procedures to benefit patients.

Both procedures integrated in this study were NVBG, but they had some differences. The lightbulb procedure can expose most of the femoral head, allowing the operator to remove necrotic bone thoroughly and fill it with autologous or allogeneic bone under direct vision [17, 32]. Although this procedure has the advantage of performing thorough debridement of necrotic bone, the massive removal of necrotic bone, especially within the anterolateral column, may reduce the mechanical stability of the femoral head to some degree and increase the risk of hip preservation failure. The Phemister procedure can provide effective mechanical support and better osseointegration for the femoral head by removing necrotic bone through one or two core decompression tracks and transplanting cancellous bone and long, straight fibula [18, 28]. This procedure has the advantage of being technically straightforward, extra-articular, and reproducible [30]. However, it has a limited scope and extent of clearance of necrotic bone, and can only clear the track and the necrotic bone in the vicinity of the track. The uneven density of the implanted healthy bone and the necrotic bone results in intra-femoral capsular degeneration, which affects long-term efficacy.

In this study, we applied radiomics to hip preservation. We screened a total of four radiomics features related to efficacy and five related to NVBG procedures, which we divided into the following two categories: (1) Shape feature describing the 3D size and shape of the ROI-Elongation, it shows the relationship between the two largest principal components in the ROI shape, and (2) Texture features-GLCM and GLDM, including MCC for complexity of the texture, Dependence Non-Uniformity Normalized for the similarity of dependence throughout the image, Mean, Joint Average, and Median for gray level intensity within the ROI. These radiomics features reflect the correlation between the extent, size, and local signal intensity of the necrotic area and efficacy of NVBG & NVBG procedures at the microscopic level.

Roger [33] found that eliminating risk factors significantly improved the survival rate for conservative treatment of ONFH. Zhu [8] proved that patients with glucocorticoid-induced osteonecrosis of the femoral head (GA-ONFH) who continued glucocorticoids treatment postoperatively had a significantly increased risk of femoral head collapse (≥ 3 mm). Consistent with these results, our study also found that continued postoperative exposure to risk factors was statistically different in both univariate and multivariate analyses (*P* < 0.05) and was an independent prognostic factor affecting hip preservation outcomes. Furthermore, univariate and multivariate analysis in our study found that exposure to risk factors postoperatively was an independent clinical predictor of NVBG efficacy (*P* < 0.05), as eliminating exposure to risk factors postoperatively improves the success rate of NVBG through the nomogram constructed from the C-R model. Depending on the type of primary disease, risk factors are classified as trauma, corticosteroid use, excessive alcohol consumption, and idiopathic cause. Therefore, we recommend that patients with GA-ONFH undergo the procedure after the primary disease has been controlled and corticosteroids are no longer used, patients with traumatic ONFH should be protected from high-risk activities that are prone to falls and fractures, patients with excessive alcohol consumption should abstain from alcohol completely, and patients with idiopathic ONFH should also be educated and have their habits adjusted to avoid exposure to risk factors known to cause ONFH.

JIC is an independent clinical predictive variable associated with the efficacy and choice of NVBG procedures, reflecting the extent of necrosis and the degree of involvement of the lateral column. The extent and location of necrosis of the femoral head are recognized factors in the prognosis of the femoral head, and the integrity of the anterolateral column plays a vital role in maintaining the function of the femoral head [34, 35]. Previous studies have concluded that patients with necrosis of < 30%, confined to the middle of the femoral head, and collapse of < 2 mm could have better efficacy of hip preservation [36, 37]. In our study, we found that the risk of hip preservation failure was higher in patients with type C2 than in those with type B and C1, which may be related to the excessive extent of the necrotic area and the greater involvement of the lateral column. Therefore, patients whose necrotic areas are mainly located in the middle and medial columns of the femoral head, while the lateral columns are more intact, will benefit more from hip preservation treatment, and the Phemister procedure should be preferred in the choice of procedure. Otherwise, the lightbulb procedure will be a better choice.

Of the three models constructed based on the above predictive variables, the C-R model outperformed the clinical model and the Radiomics model, with higher specificity and sensitivity both in the training cohort and validation cohort. Radiomics refers to the comprehensive quantification of the necrotic area by applying massive quantitative imaging features at the microscopic level, which may reflect changes in the femoral head at the cellular and genetic levels, and provides more detailed information on the necrotic area and microenvironment that are complementary to visual features [38, 39]. In contrast, the clinical predictive variables provide a macroscopic assessment of the physical condition, lifestyle, and laboratory tests of patients. The C-R model integrated the strengths of both and analyzed multimodally with the combination of partial and integral, micro, and macro, which significantly improves predictive accuracy and outperforms models with a single predictive variable. Moreover, the C-R model was visualized in the form of a nomogram to quantify the predictive variables, enabling early identification of patients with better efficacy and guiding the choice of NVBG procedures.

However, our study has several limitations: (1) This study was a retrospective single-center design, resulting in a small sample size and limited variety of surgical procedures, so multi-center, prospective, and large sample validation should be considered in future studies; (2) Our nomograms weren’t validated on an independent, external validation cohort, which would influence the predictive performance of the models. Furthermore, nomograms represent static models or algorithms and cannot easily be updated, a single and static graph is difficult to summarize the modern predictive modeling techniques and algorithm developments [40], Therefore, independent and external validation cohorts are required to verify the accuracy of the nomograms, and multiple forms of model visualization, including dynamic nomograms, are also to be explored in future research; (3) There is no suitable algorithm for the automatic segmentation of the necrotic area of the femoral head, so this study used an interactive method to manually delineate the ROI, which inevitably leads to time-consuming and errors from different operators. Convolutional neural network can be applied to improve efficiency and reduce artificial errors subsequently.

## Conclusion

In this study, we developed models using CT radiomics and clinical predictors to predict the efficacy and guide the choice of procedures in NVBG, which were visually and quantified by nomograms. Our proposed approach can be easily integrated into the clinical setting and widely used as a practical tool to predict the efficacy of NVBG preoperatively for suitable patient selection. This approach allows for the development of individualized and differentiated treatment plans without additional healthcare expenses.

### Electronic supplementary material

Below is the link to the electronic supplementary material.

Supplementary Material 1

## Data Availability

The datasets used and/or analyzed during the current study are available from the corresponding author on reasonable request.
