# Supplementary material for: Predicting efficacy and guiding procedure choice in non-vascularized bone grafting: a CT Radiomics and clinical predictor approach
Source: BMC Musculoskelet Disord. 2023 Dec 11;24:959. doi: 10.1186/s12891-023-07095-1 (PMC10712171; doi:10.1186/s12891-023-07095-1)
Supplement: Supplementary file 1 — Supplementary Material 1 [file 12891_2023_7095_MOESM1_ESM.docx]

Table S1 Clinical characteristics of patients underwent NVBG in the training and validation cohort

| Variables | Training cohort (n=130) | | Validation cohort (n=52) | | P |
| --- | --- | --- | --- | --- | --- |
|  | Success | Failure | Success | Failure |  |
| Gender (n) |  |  |  |  | 0.445 |
| Male | 64 | 35 | 38 | 5 |  |
| Female | 21 | 10 | 5 | 4 |  |
| Affected side (n) |  |  |  |  | 0.906 |
| Left | 43 | 30 | 24 | 4 |  |
| Right | 42 | 15 | 19 | 5 |  |
| Etiology (n) |  |  |  |  | 0.341 |
| Steroid | 38 | 18 | 20 | 6 |  |
| Alcohol | 14 | 11 | 7 | 2 |  |
| Trauma | 31 | 14 | 12 | 1 |  |
| Idiopathic | 2 | 2 | 4 | 0 |  |
| Exposure (n) |  |  |  |  | 0.429 |
| Yes | 16 | 26 | 8 | 5 |  |
| No | 69 | 19 | 35 | 4 |  |
| ARCO stage (n) |  |  |  |  | 0.677 |
| Stage-Ⅱ A | 62 | 27 | 33 | 6 |  |
| Stage-Ⅱ B | 21 | 13 | 8 | 3 |  |
| Stage-Ⅲ | 2 | 5 | 2 | 0 |  |
| JIC classification (n) |  |  |  |  | 0.866 |
| Type-B | 22 | 2 | 10 | 0 |  |
| Type-C1 | 48 | 23 | 24 | 6 |  |
| Type-C2 | 15 | 20 | 9 | 3 |  |
| Age(years, mean±SD) | 38.48±11.70 | 42.36±11.29 | 37.67±11.69 | 44.11±8.99 | 0.588 |
| Onset time(months, mean±SD) | 5.16±6.99 | 5.41±4.40 | 6.10±11.10 | 2.63±1.69 | 0.839 |
| BMI(kg/m^2^, mean±SD) | 23.34±2.60 | 23.51±2.52 | 23.43±2.49 | 23.31±2.22 | 0.986 |
| Harris preoperative | 69.34±9.37 | 67.60±12.32 | 69.07±9.88 | 71.77±10.41 | 0.637 |
| D-D(mg/L, mean±SD) | 0.49±0.91 | 0.67±0.81 | 0.47±0.36 | 0.41±0.16 | 0.473 |
| N(%, mean±SD) | 61.96±10.35 | 60.69±9.97 | 62.55±7.06 | 59.86±22.42 | 0.742 |
| ALP(U/L, mean±SD) | 90.45±23.73 | 87.07±27.30 | 94.69±21.78 | 90.56±30.35 | 0.128 |
| AFU(U/L, mean±SD) | 18.59±7.73 | 17.54±7.32 | 16.85±5.57 | 19.10±5.45 | 0.399 |

Table S2 Clinical characteristics of patients underwent NVBG with Phemister or lightbulb procedure in the training and validation cohort

| Variables | Training cohort (n=92) | | Validation cohort (n=36) | | P |
| --- | --- | --- | --- | --- | --- |
|  | Phemister | lightbulb | Phemister | lightbulb |  |
| Gender (n) |  |  |  |  | 0.285 |
| Male | 56 | 20 | 18 | 8 |  |
| Female | 11 | 5 | 7 | 3 |  |
| Affected side (n) |  |  |  |  | 0.356 |
| Left | 38 | 13 | 11 | 5 |  |
| Right | 29 | 12 | 14 | 6 |  |
| Etiology (n) |  |  |  |  | 0.869 |
| Steroid | 30 | 11 | 13 | 4 |  |
| Alcohol | 12 | 4 | 2 | 3 |  |
| Trauma | 22 | 8 | 10 | 3 |  |
| Idiopathic | 3 | 2 | 0 | 1 |  |
| Exposure (n) |  |  |  |  | 0.900 |
| Yes | 11 | 7 | 3 | 3 |  |
| No | 56 | 18 | 22 | 8 |  |
| ARCO stage (n) |  |  |  |  | 0.543 |
| Stage-Ⅱ A | 52 | 18 | 19 | 6 |  |
| Stage-Ⅱ B | 13 | 7 | 6 | 3 |  |
| Stage-Ⅲ | 2 | 0 | 0 | 2 |  |
| JIC classification (n) |  |  |  |  | 0.872 |
| Type-B | 22 | 0 | 10 | 0 |  |
| Type-C1 | 34 | 18 | 12 | 8 |  |
| Type-C2 | 11 | 7 | 3 | 3 |  |
| Age(years, mean±SD) | 39.09±11.57 | 35.28±11.56 | 37.72±12.26 | 40.64±11.30 | 0.809 |
| Onset time(months, mean±SD) | 5.45±10.30 | 3.72±3.32 | 6.64±7.96 | 7.00±6.21 | 0.295 |
| BMI(kg/m^2^, mean±SD) | 23.82±2.29 | 22.98±3.33 | 23.15±1.99 | 22.04±2.93 | 0.122 |
| Harris preoperative | 69.43±9.91 | 67.48±8.90 | 73.16±6.49 | 63.27±11.08 | 0.510 |
| D-dimer(mg/L, mean±SD) | 0.49±1.01 | 0.52±0.41 | 0.46±0.39 | 0.44±0.21 | 0.746 |
| N(%, mean±SD) | 61.03±8.84 | 63.73±7.68 | 63.59±10.92 | 62.19±12.09 | 0.448 |
| ALP(U/L, mean±SD) | 92.61±27.01 | 99.48±31.40 | 95.40±24.82 | 89.45±17.31 | 0.866 |
| AFU(U/L, mean±SD) | 17.77±7.50 | 18.89±8.20 | 17.34±5.21 | 18.98±6.21 | 0.871 |
